# Dietary *L*-arginine supplementation reduces lipid accretion by regulating fatty acid metabolism in Nile tilapia (*Oreochromis niloticus*)

**DOI:** 10.1186/s40104-020-00486-7

**Published:** 2020-08-14

**Authors:** Senlin Li, Yunchang Zhang, Ning Liu, Jingqing Chen, Lina Guo, Zhaolai Dai, Chao Wang, Zhenlong Wu, Guoyao Wu

**Affiliations:** 1 State Key Laboratory of Animal Nutrition, Department of Animal Nutrition and Feed Science, China Agricultural University, Beijing, 100193 China; 2 College of Biological Sciences, China Agricultural University, Beijing, 100193 China; 3 Department of Animal Science, Texas A&M University, College Station, TX 77843 USA

**Keywords:** Fatty acid composition, *L*-arginine, Lipid metabolism, Tilapia

## Abstract

**Background:**

Excessive white fat accumulation in humans and other animals is associated with the development of multiple metabolic diseases. It is unknown whether dietary *L*-arginine supplementation reduces lipid deposition in high fat diet-fed Nile tilapia (*Oreochromis niloticus*).

**Results:**

In the present study, we found that dietary supplementation with 1% or 2% arginine decreased the deposition and concentration of fats in the liver; the concentrations of triglycerides, low-density lipoprotein, total cholesterol, and high-density lipoprotein in the serum; and the diameter of adipocytes in intraperitoneal adipose tissue. Compared with the un-supplementation control group, the hepatic activities of alanine aminotransferase, aspartate aminotransferase, and lactate dehydrogenase, and hepatic concentration of malondialdehyde were reduced but these for catalase and superoxide dismutase were enhanced by dietary supplementation with 2% arginine. Arginine supplementation reduced the total amounts of monounsaturated fatty acids, while increasing the total amounts of n-3 and n-6 polyunsaturated fatty acids in the liver. These effects of arginine were associated with reductions in mRNA levels for genes related to lipogenesis (sterol regulatory element-binding protein-1, acetyl-CoA carboxylase *α,* stearoyl-CoA desaturase, and fatty acid synthase) but increases in mRNA levels for genes involved in fatty acid *β*-oxidation (carnitine palmitoyltransferase 1*α* and peroxisome proliferator-activated receptor *α*). In addition, hepatic mRNA levels for Δ4 fatty acyl desaturase 2 and elongase 5 of very long-chain fatty acids were enhanced by arginine supplementation.

**Conclusion:**

These results revealed that dietary L-arginine supplementation to tilapia reduced high fat diet-induced fat deposition and fatty acid composition in the liver by regulating the expression of genes for lipid metabolism.

## Introduction

*L*-Arginine (Arg) is a precursor for the synthesis of biologically important molecules, including nitric oxide (NO), polyamines, creatine, agmatine, proline, and glutamate [1]. In addition to serving as a substrate for protein synthesis, Arg is a crucial regulator of various biological processes, including intestinal growth and development, embryonic implantation and survival, gene expression, and immune response in mammals [2, 3]. It has been reported that dietary supplementation with Arg decreases body white fat deposition, promotes the oxidation of long-chain fatty acids, and decreases the synthesis of triacylglycerols *de novo* in mammalian models [4, 5]. However, few studies have been conducted to investigate the effects of dietary Arg on the lipid metabolism in fish.

Increasing the provision of dietary lipids can spare some dietary protein and promote body weight gains in animals, including fish [6]. However, excessive energy intake from fats is associated with white fat accumulation in various tissues, such as the white adipose tissue, skeletal muscle, liver, pancreas, and heart, leading to the development of multiple disorders, including gastrointestinal abnormalities, cardiovascular disease, metabolic syndrome, reduced food intake, and reduced efficiency in the utilization of nutrients for protein gain [7, 8]. Both clinical and experimental studies have shown that the deposition of fats in the liver (also known as fatty liver) modifies fatty acid profiles and affects the function of both the liver and whole body in animals [9, 10]. A high hepatic concentration of fatty acids is positively correlated with the occurrence of the fatty liver in animals (including fish) [11, 12].

Nile tilapia (*Oreochromis niloticus*) is a main fish species in the aquaculture industry around the world due to its rapid growth rate and resistance to various environmental conditions [13]. With the development of intensive farming, high incidences of the fatty liver and related mortality due to an excessive intake of high-fat diets hinder the production of the fish as a source of high-quality protein for human consumption [6]. Also, it has been proposed that high fat diet-induced fatty liver of tilapia might be a useful model for research on nutrition and fat accumulation [6, 13]. However, nutritional strategies to reduce the detrimental effects of the fatty liver are not available. In this regard, it is noteworthy that dietary supplementation with Arg has been reported to reduce obesity in rats [14] and improve metabolic profiles in humans [15]. The present study was conducted to test the hypothesis that Arg supplementation to tilapia might reduce lipid accretion in the liver.

## Methods

### Animals and experimental design

This study was approved by the Institutional Animal Care and Use Committee of China Agricultural University. After a two-week period of adaptation, a total of 180 tilapia (genetically improved farmed tilapia) with a similar body weight (2.35 ± 0.01 g) were assigned randomly into one of three groups. Each group consisted of 3 tanks, with 20 fish per tank (150 L; 50 cm × 50 cm × 60 cm). During the 56-day feeding experiment, the fish were fed a commercial diet (Huaqin Agro-Tech Co Ltd., China) un-supplemented or supplemented with 1% or 2% Arg. The dose of Arg supplemented in the present study was based on our pilot study showing that adding 1–2% Arg to the basal diet reduced lipid accumulation in tilapia. The ingredients, as well as the composition of amino acids and fatty acids in the diets are shown in Supplemental Table 1, Supplemental Table 2, and Supplemental Table 3, respectively. Ala and starch were used to formulate iso-nitrogenous and iso-caloric diets [14]. All the fish were fed 3 times daily to apparent satiation. The quality, temperature, dissolved oxygen, pH, nitrites, sulfide, and ammonia content in the aquatic water were recorded during the whole experimental period (28 °C, 6.5 mg/L, 8, 0.039 mg/L, < 0.05 mg/L, and < 0.1 mg/L, respectively). Sera and tissues, including the liver and intraperitoneal adipose tissue, were collected and stored at − 80 °C until analysis.

### Sampling

After 24 h of fasting, all fish were anaesthetized with MS-222 and their body weights were measured. Six fish from each tank were randomly selected for blood sampling from the caudal vein. The serum was separated by centrifugation at 825×*g* for 10 min at 4 °C, and then was stored at − 80 °C for later analysis. Fifteen fish from each tank were dissected, and hepatopancreas, intraperitoneal fat, and spleen of the fish were quickly removed, weighed and stored at − 80 °C until analyzed.

### Analyses of fatty acids

The fatty acid composition of diets and tissues were determined by gas chromatography (Hewlett-Packard HP6890 GC system) equipped with a Chrompack Capillary Column (CP-Sil 88 column), as previously described [16]. Briefly, diets and tissues were homogenized (Bead Ruptor 12; Omni International) for 1 min and an alique of 0.5 g was sampled for fatty acid extraction. Liver tissues were extracted with chloroform:methanol (2:1, v:v). The extracted fat was saponified with methanolic potassium hydroxide and methylated with methanol solution (0.4 mol/L potassium hydroxide). Fatty acid methyl esters were determined by gas chromatography and results are expressed as a fraction of the total amount of fatty acid methyl esters.

### Analyses of amino acids

The concentrations of amino acids in diets were analyzed by HPLC methods as previously described [17], except that a model of Waters 2690 (Waters Chromatography Division, Milford, MA, USA) was used for the separation and quantification of amino acids.

### Biochemical measurements

Sera and liver tissues were collected for biochemical analyses. Lipids in the liver were measured by diethyl ether extraction using the Soxhlet method [18]. The activities of alanine aminotransferase (ALT), aspartate aminotransferase (AST), superoxide dismutase (SOD), catalase (CAT), and lactate dehydrogenase (LDH) were measured using commercial kits (Jiancheng Biotechnology Co., Nanjing, China), according to the manufacturer’s instructions. The concentrations of non-esterified free fatty acids (NEFA), triglycerides (TG), total cholesterol (TC), low-density lipoprotein cholesterol (LDL), high-density lipoprotein cholesterol (HDL), and malondialdehyde (MDA) were measured by using commercial kits (Jiancheng Biotechnology Co., Nanjing, China), according to the manufacturer’s protocols.

### Histological analyses

The liver and intraperitoneal adipose tissues were fixed in 4% paraformaldehyde for 24 h, followed by dehydration and embedding in paraffin. Five micrometer-thick tissue sections were sectioned using a microtome (Thermo Fisher Scientific, San Jose, CA, USA) and were subjected to H&E staining. Histological alteration was observed and photographed by using an inverted microscope (OPTEC, China). The diameter of adipocytes was determined by using a microscope equipped with TSView 7 software (Tucsen, China).

### Quantitative real-time PCR

Total RNA was isolated from the liver with the use of the Trizol reagent (CWBio Biotech Co., Beijing), and was reverse-transcribed into cDNA by using the PrimeScript RT reagent (Takara, Japan), according to the manufacturer’s procedures. Quantitative real-time PCR analysis was performed by the SYBR green method and the Applied Biosystems 7500 real-time PCR system. Tilapia-specific primers were designed according to sequences of the genes and were synthesized by Sangon Biotech. The specificity of primers was examined by Primer-BLAST tool (https://www.ncbi.nlm.nih.gov/tools/primer-blast) and confirmed by single peaks in the dissociation curves. The EF1α gene was used as an internal control. The primer sequences were listed in Supplemental Table 4. The qPCR data were analyzed by using the 2^−ΔΔCt^ method.

### Statistical analysis

The results are presented as means ± SEM. Data were analyzed by one-way ANOVA. Differences between means were determined by the Duncan multiple comparisons test. All statistical analyses were performed by the SPSS statistical software (SPSS for Windows, version 25.0). *P* < 0.05 was taken to indicate statistical significance.

## Results

### Growth performance and organ index of tilapia

As shown in Table 1, the initial body weight of tilapia did not differ (*P* > 0.05) among the 3 treatment groups. Dietary supplementation with 1% or 2% Arg did not affect (*P* > 0.05) the final body weight, weight gain, specific growth rate, feed intake, or feed conversion ratio, as compared with the control group. Interestingly, supplementation with 1% or 2% Arg significantly reduced body condition scores and hepatopancreas index, while significantly enhancing spleen index. In addition, supplementation with 2% Arg, but not 1% Arg, significantly reduced (*P* < 0.05) the intraperitoneal fat index.

**Table 1:** Effects of dietary supplementation with 1% Arg, or 2% Arg for 56 days on growth performance and organ index of tilapia^1^

Items	Control	1%Arg	2%Arg	*P*-value
Initial body weight, g	2.33 ± 0.02	2.35 ± 0.04	2.36 ± 0.03	0.691
Final body weight, g	67.47 ± 4.53	68.64 ± 3.56	68.27 ± 4.19	0.979
Weight gain, g	65.15 ± 2.83	66.02 ± 3.00	66.43 ± 2.48	0.957
Specific growth rate, %/d	5.96 ± 0.08	5.99 ± 0.07	5.99 ± 0.06	0.970
Feed conversion ratio	0.85 ± 0.05	0.86 ± 0.05	0.87 ± 0.06	0.985
Feed intake, % of body weight/d	2.83 ± 0.17	2.87 ± 0.17	2.88 ± 0.19	0.979
Body condition factor, g/cm^3^ of body length	3.52 ± 0.03^a^	3.33 ± 0.01^b^	3.29 ± 0.02^b^	0.000
Liver index, %	1.90 ± 0.07^a^	1.61 ± 0.26^b^	1.28 ± 0.23^c^	0.000
Spleen index, %	0.30 ± 0.02^c^	0.45 ± 0.03^b^	0.68 ± 0.03^a^	0.000
Intraperitoneal fat index, %	2.03 ± 0.11^a^	1.78 ± 0.18^ab^	1.45 ± 0.06^b^	0.041

^1^Values are means ± SEM, *n* = 3 tanks per group. Means without a common letter differ, *P* < 0.05. Body condition factor = body weight (g) × 100 / body length^3^ (cm); Feed conversion ratio = amount of feed intake (g) / weight gain (g); Feed intake = 100 × total amount of the feed consumed (g) / [(initial body weight (g) + final body weight (g)) / 2]/ days; Organ index = organ weight (g) × 100 / body weight (g); Specific growth rate = (Ln final weight (g) – Ln initial weight (g)) × 100 / days; Weight gain = final weight (g) – initial weight (g)

### Histology of the liver and intraperitoneal adipose tissue

**Fig. 1:**
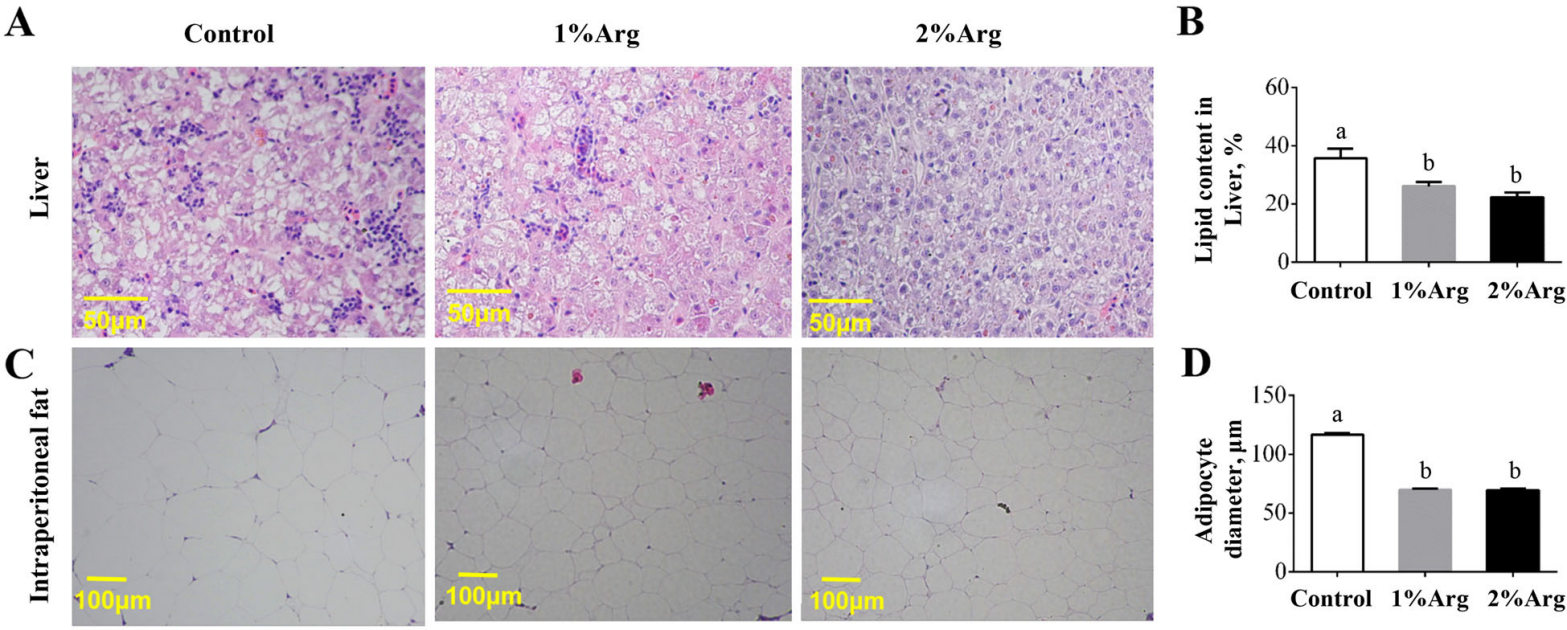
Histological alteration (**a**), lipid content of the liver (**b**), representative images of intraperitoneal adipose tissue (**c**), and the diameter of adipocytes in intraperitoneal adipose tissue (**d**) of tilapia fed a commercial diet un-supplemented (control) or supplemented with 1% or 2% Arg. Values are means ± SEM, *n* = 3 tanks per group. Means without a common letter differ, *P* < 0.05

Anatomic examination of tilapia at the end of the experiment showed that fish in the control diet had a large amount of intraperitoneal adipose tissue and pale livers, which were alleviated by Arg supplementation (Supplemental Fig. 1). Histological analysis showed that supplementation with 1% or 2% Arg significantly decreased (*P* < 0.05) lipid content (Fig. 1a-b) in the liver of tilapia. Further study showed that Arg supplementation significantly reduced the diameter of adipocytes in intraperitoneal adipose tissue (Fig. 1c-d), as compared with the control group, indicating a regulatory effect of Arg on lipid accretion in the liver.

### Serum and hepatic lipids and lipoproteins

**Fig. 2:**
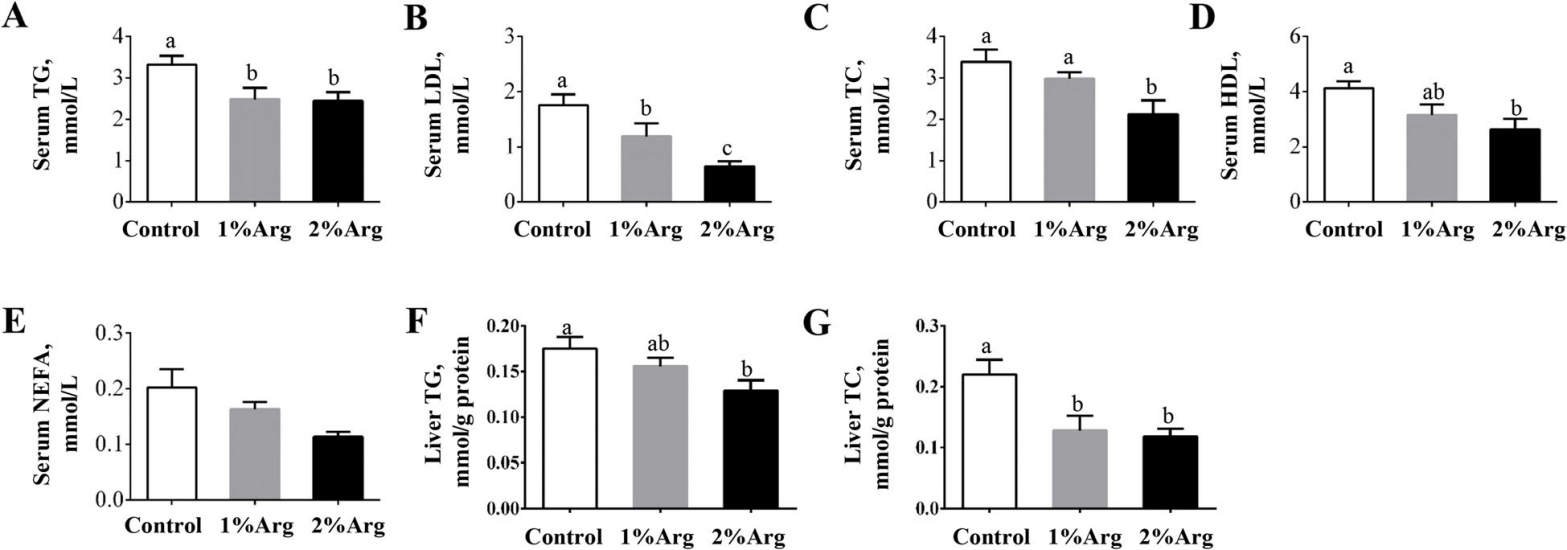
The concentrations of serum TG (**a**), LDL (**b**), TC (**c**), HDL (**d**), and NEFA (**e**), and hepatic concentrations of TG (**f**) and TC (**g**) in tilapia fed a commercial diet un-supplemented (control) or supplemented with 1% or 2%Arg. Values are means ± SEM, *n* = 3 tanks per group. Means without a common letter differ, *P* < 0.05. HDL, high-density lipoprotein cholesterol; LDL, low-density lipoprotein cholesterol; NEFA, non-esterified fatty acids; TC, total cholesterol; TG, triglyceride

Compared with the control, dietary supplementation with 1% or 2% Arg significantly reduced (*P* < 0.05) the concentrations of both TG and LDL in serum (Fig. 2a-b). The concentrations of TC and HDL in the serum of the 2% Arg group were significantly decreased (*P* < 0.05) by 2%, compared with the control group (Fig. 2c-d). In contrast, serum NEFA was not affected (*P* > 0.05) by Arg supplementation (Fig. 2e). Additionally, the concentrations of both TG and TC (Fig. 2f-g) in the liver were significantly decreased (*P* < 0.05) dietary supplementation with 2% Arg, but those for TC were decreased (*P* > 0.05) by supplementation with 1% Arg.

### Fatty acid composition of liver and intraperitoneal adipose tissue

As shown in Table 2, Arg supplementation had no effect (*P* > 0.05) on the content of total SFA in the liver. As compared with the control group, dietary supplementation with 1% or 2% Arg significantly decreased (*P* < 0.05) the proportion of total MUFAs (*P* < 0.05), while significantly increasing (*P* < 0.05) the proportions of total PUFAs and total n-3 PUFAs in the liver. In addition, supplementation with 2% Arg, but not 1% Arg, significantly augmented (*P* < 0.05) the proportion of total n-6 PUFAs and the ratio of n-6:n-3 PUFAs in the liver of tilapia (*P* < 0.05).

**Table 2:** Fatty acid composition (% of total fatty acids) in the liver of tilapia fed the basal diet un-supplemented (control), or supplemented with 1% Arg, or 2% Arg for 56 days^1^

Fatty acid	Control	1%Arg	2%Arg	*P-*value
C12:0	0.03 ± 0.00	0.02 ± 0.00	0.04 ± 0.01	0.055
C14:0	1.70 ± 0.06^a^	1.44 ± 0.02^b^	1.49 ± 0.05^b^	0.033
C15:0	0.07 ± 0.01^b^	0.07 ± 0.00^b^	0.10 ± 0.00^a^	0.006
C16:0	21.26 ± 0.16	21.20 ± 0.29	21.13 ± 0.17	0.935
C17:0	0.23 ± 0.01^b^	0.23 ± 0.00^b^	0.29 ± 0.01^a^	0.003
C18:0	11.61 ± 0.09	11.40 ± 0.23	12.06 ± 0.27	0.267
C20:0	0.21 ± 0.01^a^	0.18 ± 0.00^b^	0.15 ± 0.01^b^	0.014
C21:0	1.55 ± 0.11	1.90 ± 0.04	1.74 ± 0.06	0.084
C22:0	0.20 ± 0.01^a^	0.16 ± 0.00^b^	0.12 ± 0.00^c^	0.004
C23:0	0.13 ± 0.00	0.12 ± 0.00	0.11 ± 0.00	0.086
C24:0	0.57 ± 0.05	0.52 ± 0.01	0.55 ± 0.01	0.639
∑ SFAs	37.57 ± 0.23	37.23 ± 0.47	37.77 ± 0.33	0.694
C14:1n-5	0.02 ± 0.00	0.02 ± 0.00	0.01 ± 0.00	0.422
C16:1n-7	1.56 ± 0.05^a^	1.69 ± 0.07^a^	1.26 ± 0.05^b^	0.011
C18:1n-9	36.49 ± 0.42^a^	33.57 ± 0.39^b^	27.18 ± 0.09^c^	0.000
C20:1n-9	1.65 ± 0.22	1.87 ± 0.01	1.20 ± 0.02	0.062
C22:1n-9	0.05 ± 0.00^b^	0.09 ± 0.00^a^	0.05 ± 0.00^b^	0.000
C24:1n-9	0.12 ± 0.01^b^	0.10 ± 0.00^b^	0.23 ± 0.01^a^	0.000
∑ MUFAs	39.89 ± 0.61^a^	37.33 ± 0.43^b^	29.94 ± 0.11^c^	0.000
C18:3n-3	0.76 ± 0.04	0.76 ± 0.03	0.97 ± 0.06	0.073
C20:3n-3	0.27 ± 0.01^b^	0.26 ± 0.01^b^	0.39 ± 0.01^a^	0.001
C20:5n-3	0.04 ± 0.00^b^	0.04 ± 0.00^b^	0.07 ± 0.00^a^	0.001
C22:6n-3	3.23 ± 0.07^c^	4.05 ± 0.04^b^	7.08 ± 0.23^a^	0.000
∑ n-3 PUFAs	4.30 ± 0.11^c^	5.11 ± 0.08^b^	8.51 ± 0.18^a^	0.000
C18:2n-6	13.28 ± 0.43^b^	15.32 ± 0.72^ab^	16.48 ± 0.24^a^	0.028
C20:3n-6	1.68 ± 0.08	1.48 ± 0.02	1.54 ± 0.06	0.259
C20:4n-6	3.25 ± 0.13^b^	3.49 ± 0.05^b^	5.73 ± 0.14^a^	0.000
C22:2n-6	0.03 ± 0.01	0.04 ± 0.00	0.04 ± 0.00	0.702
∑ n-6 PUFAs	18.24 ± 0.30^b^	20.33 ± 0.79^b^	23.78 ± 0.42^a^	0.003
∑ PUFAs	22.55 ± 0.39^c^	25.44 ± 0.86^b^	32.29 ± 0.40^a^	0.000
n-6:n-3	4.24 ± 0.06^a^	3.97 ± 0.10^a^	2.80 ± 0.09^b^	0.000

^1^Values are means ± SEM, *n* = 3 tanks per group. Means without a common letter differ, *P* < 0.05. MUFAs, monounsaturated fatty acids; n-6:n-3, n-6 PUFAs:n-3 PUFAs; PUFAs, polyunsaturated fatty acids; SFAs, saturated fatty acids

As shown in Table 3, total SFAs of intraperitoneal adipose tissue did not differ (*P* > 0.05) among the 3 groups of fish. The proportion of total MUFAs in intraperitoneal adipose tissue was significantly reduced (*P* < 0.05) by dietary supplementation with 2% Arg, but not 1% Arg. The proportion of total PUFAs in intraperitoneal adipose tissue was significantly elevated (*P* < 0.05) by dietary supplementation with 1% or 2% Arg. However, supplementation with 2% Arg, but not 1% Arg, significantly augmented (*P* < 0.05) total n-3 PUFAs and n-6 PUFAs in intraperitoneal adipose tissue. No difference in the ratio of n-6:n-3 PUFAs was detected (*P* > 0.05) among the 3 treatment groups in intraperitoneal adipose tissue.

**Table 3:** Fatty acid composition (% of total fatty acids) in the intraperitoneal fat of tilapia fed the basal diet un-supplemented (control), or supplemented with 1% Arg, or 2% Arg for 56 days^1^

Fatty acid	Control	1%Arg	2%Arg	*P*-value
C12:0	0.03 ± 0.00^b^	0.03 ± 0.00^b^	0.04 ± 0.00^a^	0.027
C14:0	1.28 ± 0.04	1.40 ± 0.03	1.25 ± 0.07	0.281
C15:0	0.10 ± 0.00	0.09 ± 0.00	0.10 ± 0.01	0.252
C16:0	16.15 ± 0.18^a^	15.59 ± 0.10^b^	16.28 ± 0.10^a^	0.046
C17:0	0.16 ± 0.01	0.15 ± 0.00	0.16 ± 0.01	0.422
C18:0	5.63 ± 0.14^a^	5.89 ± 0.13^a^	4.92 ± 0.07^b^	0.007
C20:0	0.29 ± 0.00	0.27 ± 0.00	0.29 ± 0.01	0.332
C21:0	1.64 ± 0.02	1.66 ± 0.02	1.68 ± 0.03	0.736
C22:0	0.16 ± 0.00	0.15 ± 0.00	0.17 ± 0.00	0.064
C23:0	0.09 ± 0.00	0.09 ± 0.01	0.09 ± 0.01	0.885
C24:0	0.65 ± 0.03	0.61 ± 0.01	0.60 ± 0.02	0.532
∑ SFAs	26.17 ± 0.08	25.93 ± 0.02	25.58 ± 0.20	0.089
C14:1n-5	0.02 ± 0.00	0.02 ± 0.00	0.02 ± 0.00	0.422
C16:1n-7	1.48 ± 0.04	1.53 ± 0.03	1.61 ± 0.03	0.144
C18:1n-9	30.84 ± 0.17^a^	30.28 ± 0.09^b^	28.76 ± 0.06^c^	0.000
C20:1n-9	1.25 ± 0.03	1.28 ± 0.03	1.19 ± 0.05	0.45
C22:1n-9	0.09 ± 0.01	0.09 ± 0.00	0.09 ± 0.01	0.936
C24:1n-9	0.04 ± 0.00	0.04 ± 0.00	0.04 ± 0.00	0.178
∑ MUFAs	33.72 ± 0.16^a^	33.23 ± 0.14^a^	31.72 ± 0.07^b^	0.000
C18:3n-3	2.97 ± 0.03^b^	3.46 ± 0.05^ab^	3.66 ± 0.21^a^	0.047
C20:3n-3	0.41 ± 0.00	0.43 ± 0.01	0.43 ± 0.00	0.182
C20:5n-3	0.10 ± 0.00	0.10 ± 0.00	0.11 ± 0.01	0.308
C22:6n-3	1.29 ± 0.06	1.13 ± 0.04	1.16 ± 0.07	0.324
∑ n-3 PUFAs	4.77 ± 0.05^b^	5.11 ± 0.03^ab^	5.35 ± 0.14^a^	0.027
C18:2n-6	32.61 ± 0.13^c^	33.23 ± 0.11^b^	34.97 ± 0.15^a^	0.000
C20:3n-6	1.57 ± 0.08	1.47 ± 0.03	1.42 ± 0.07	0.425
C20:4n-6	1.12 ± 0.01	0.99 ± 0.04	0.91 ± 0.06	0.092
C22:2n-6	0.05 ± 0.00	0.05 ± 0.00	0.06 ± 0.01	0.870
∑ n-6 PUFAs	35.34 ± 0.16^b^	35.73 ± 0.10^b^	37.36 ± 0.08^a^	0.000
∑ PUFAs	40.11 ± 0.13^c^	40.84 ± 0.13^b^	42.71 ± 0.17^a^	0.000
n-6:n-3	7.41 ± 0.11	6.99 ± 0.03	6.99 ± 0.19	0.175

^1^Values are means ± SEM, *n* = 3 tanks per group. Means without a common letter differ, *P* < 0.05. MUFAs, monounsaturated fatty acids; n-6:n-3, n-6 PUFAs:n-3 PUFAs; *PUFAs* Polyunsaturated fatty acids, *SFAs* Saturated fatty acids

### mRNA levels of genes related to hepatic lipid metabolism and PUFA synthesis

**Fig. 3:**
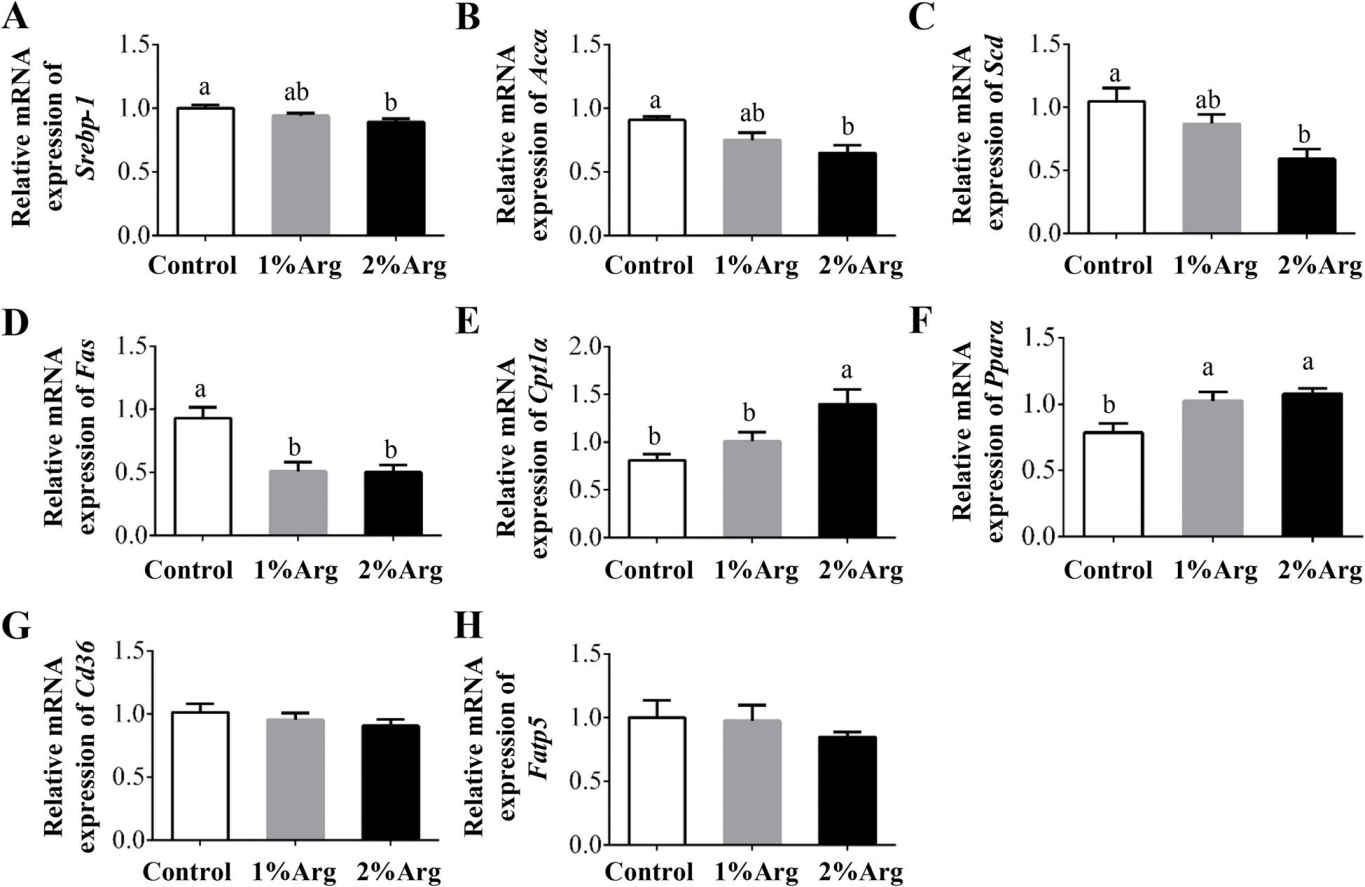
Hepatic mRNA levels for *Srebp-1* (**a**), *Accα* (**b**), *Scd* (**c**), *Fas* (**d**), *Cpt1α* (**e**), *Pparα* (**f**), *Cd36* (**g**), *Fatp5* (**h**) in tilapia fed a commercial diet un-supplemented (control) or supplemented with 1% or 2%Arg. Values are means ± SEM, *n* = 3 tanks per group. Means without a common letter differ, *P* < 0.05. *Srebp-1*, sterol regulatory element-binding protein-1; *Accα,* acetyl-CoA carboxylase; *Scd*, stearoyl-CoA desaturase; *Fas*, fatty acid synthase; *Cpt1α,* carnitine palmitoyltransferase 1*α*; *Pparα,* peroxisome proliferator-activated receptor *α*; *Cd36,* cluster determinant 36; *Fatp5,* fatty acid transport protein 5

Compared to the control group, mRNA levels for sterol regulatory element-binding protein-1 (*Srebp-1*), acetyl-CoA carboxylase *α* (*Accα*), and stearoyl-CoA desaturase (*Scd*) were significantly reduced (Fig. 3a-c), whereas those for carnitine palmitoyltransferase 1*α* (*Cpt1α*) were significantly enhanced (*P* < 0.05), by dietary supplementation with 2% Arg (Fig. 3e). Supplementation with 1% Arg had no effect (*P* > 0.05) on these variables. The mRNA level of *Fas* was significantly reduced (*P* < 0.05), but that for *Pparα* was significantly increased (*P* < 0.05) by supplementation with 1% or 2% Arg (Fig. 3d and f). Arg supplementation did not affect (*P* > 0.05) the mRNA levels for cluster determinant 36 (*Cd36*) and fatty acid transport protein 5 (*Fatp5*), two genes involved in the transport of long-chain fatty acids (Fig. 3g-h).

**Fig. 4:**
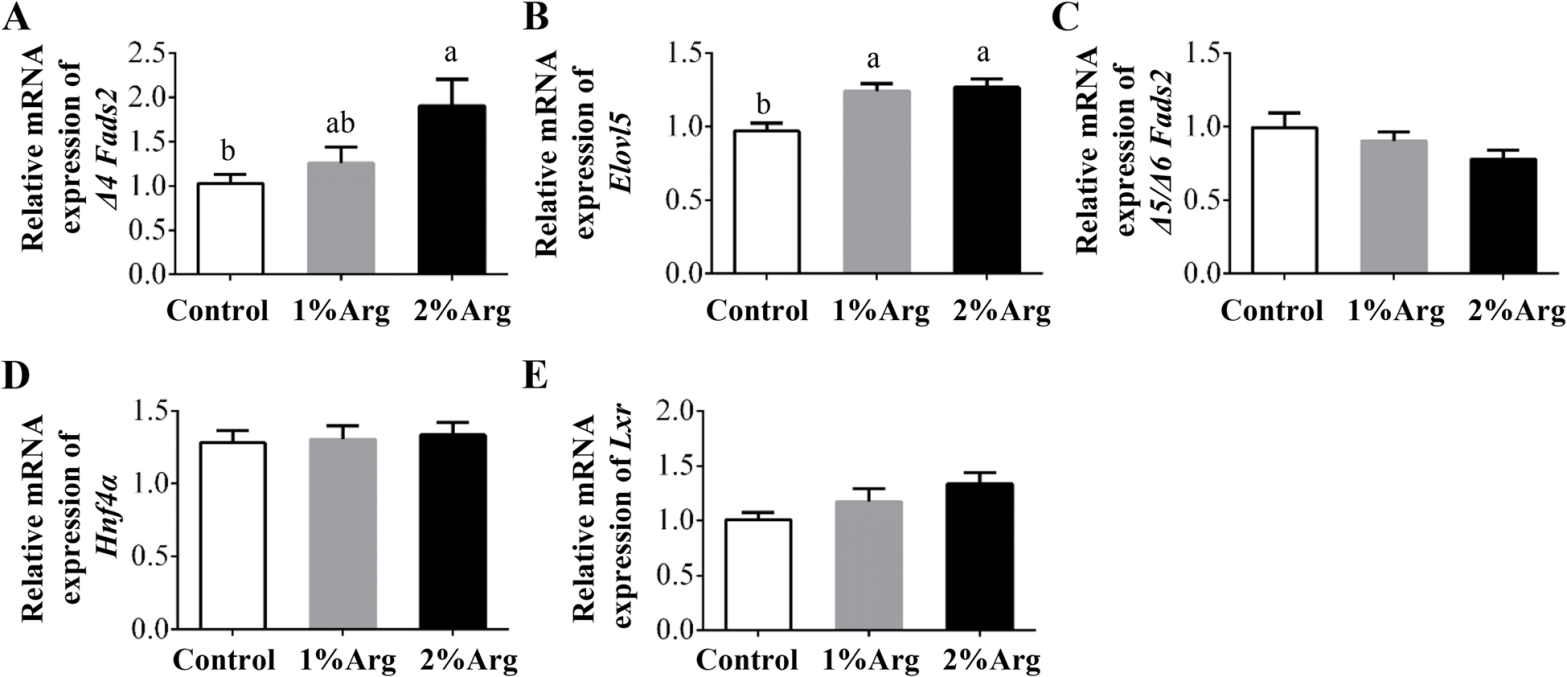
Hepatic mRNA level of *Δ4 Fads2* (**a**), *Elovl5* (**b**), *Δ5/Δ6 Fads2* (**c**), *Hnf4α* (**d**), and *Lxr* (**e**) in tilapia fed a commercial diet un-supplemented (control) or supplemented with 1% or 2%Arg. Values are means ± SEM, *n* = 3 tanks per group. Means without a common letter differ, *P* < 0.05. *Δ4 Fads2*, *Δ4* fatty acyl desaturase 2; *Δ5/Δ6 Fads2,* Δ5/Δ6 fatty acyl desaturase 2; *Elovl5,* elongase 5 of very long-chain fatty acids; *Hnf4α*, hepatocyte nuclear factor 4α; *Lxr*, liver X receptor

The mRNA level of *Δ4 Fads2* was significantly elevated (*P* < 0.05) by dietary supplementation with 2% Arg, but not 1% Arg (Fig. 4a). Supplementation with 1% or 2% Arg significantly increased (*P* < 0.05) the mRNA level of *Elovl5* in the liver (Fig. 4b). However, supplementation with 1% or 2% Arg did not affect (*P* > 0.05) mRNA levels for *Δ5/Δ6 Fads2*, hepatocyte nuclear factor 4α (*Hnf4α*), and liver X receptor (*Lxr*) (*P* > 0.05), as compared with the controls (Fig. 4c-e).

### Activities of ALT, AST and LDH in the serum and liver of tilapia

**Fig. 5:**
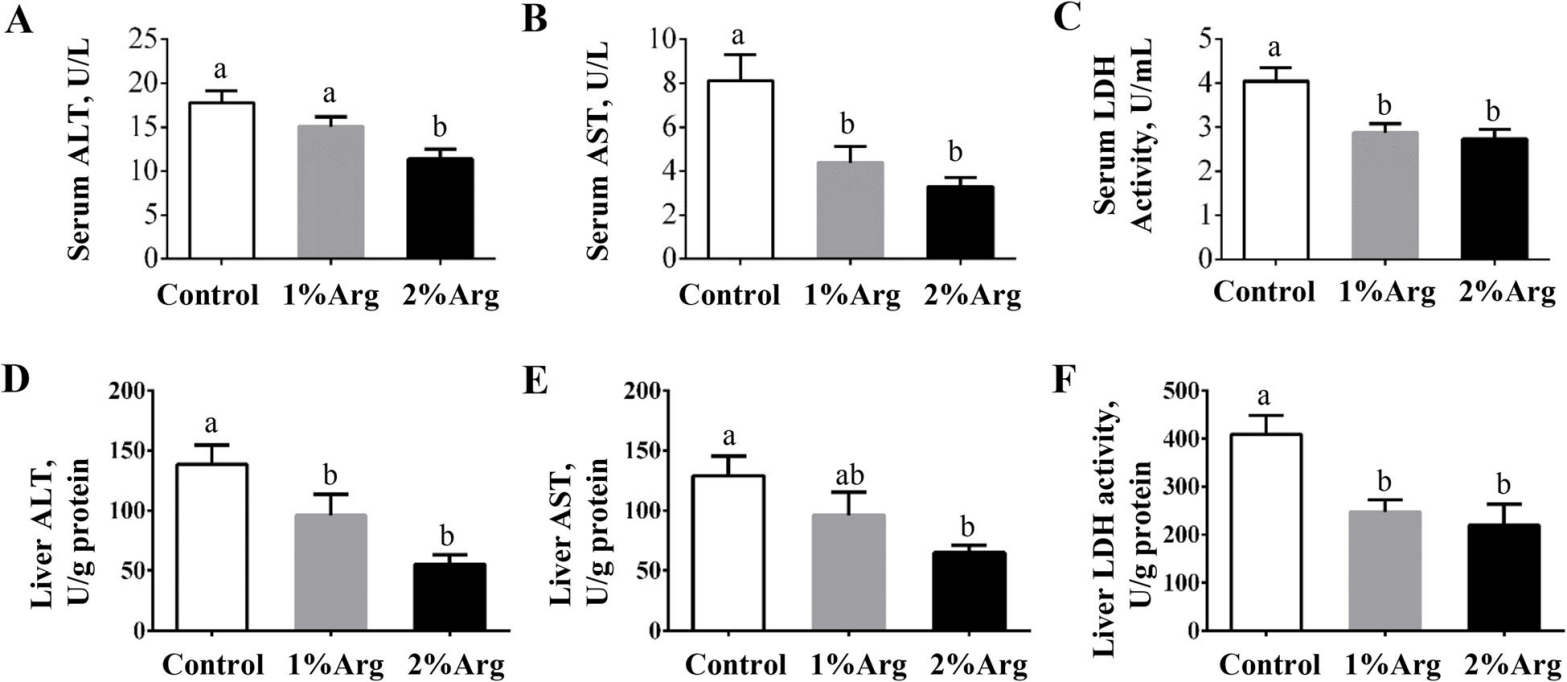
Activities of ALT (**a**), AST (**b**), LDH (**c**) in serum and of ALT (**d**), AST (**e**), LDH (**f**) in the liver of tilapia fed a commercial diet un-supplemented (control) or supplemented with 1% or 2%Arg. Values are means ± SEM, *n* = 3 tanks per group. Means without a common letter differ, *P* < 0.05. ALT, alanine aminotransferase; AST, aspartate aminotransferase; LDH, lactate dehydrogenase

In comparison to the control group, the activity of serum ALT was significantly decreased (*P* < 0.05) by dietary supplementation with 2% Arg, but not 1% Arg (Fig. 5a). Supplementation with 1% or 2% Arg significantly decreased (*P* < 0.05) the activities of serum AST and LDH (Fig. 5b-c). Additionally, the hepatic activities of ALT and LDH were significantly reduced (*P* < 0.05) by dietary supplementation with 1% or 2% Arg (Fig. 5d and f). The hepatic activity of AST was significantly reduced (*P* < 0.05) in the 2% Arg group, compared with the control group (Fig. 5e).

### Activities of CAT, SOD and MDA in the serum and liver of tilapia

**Fig. 6:**
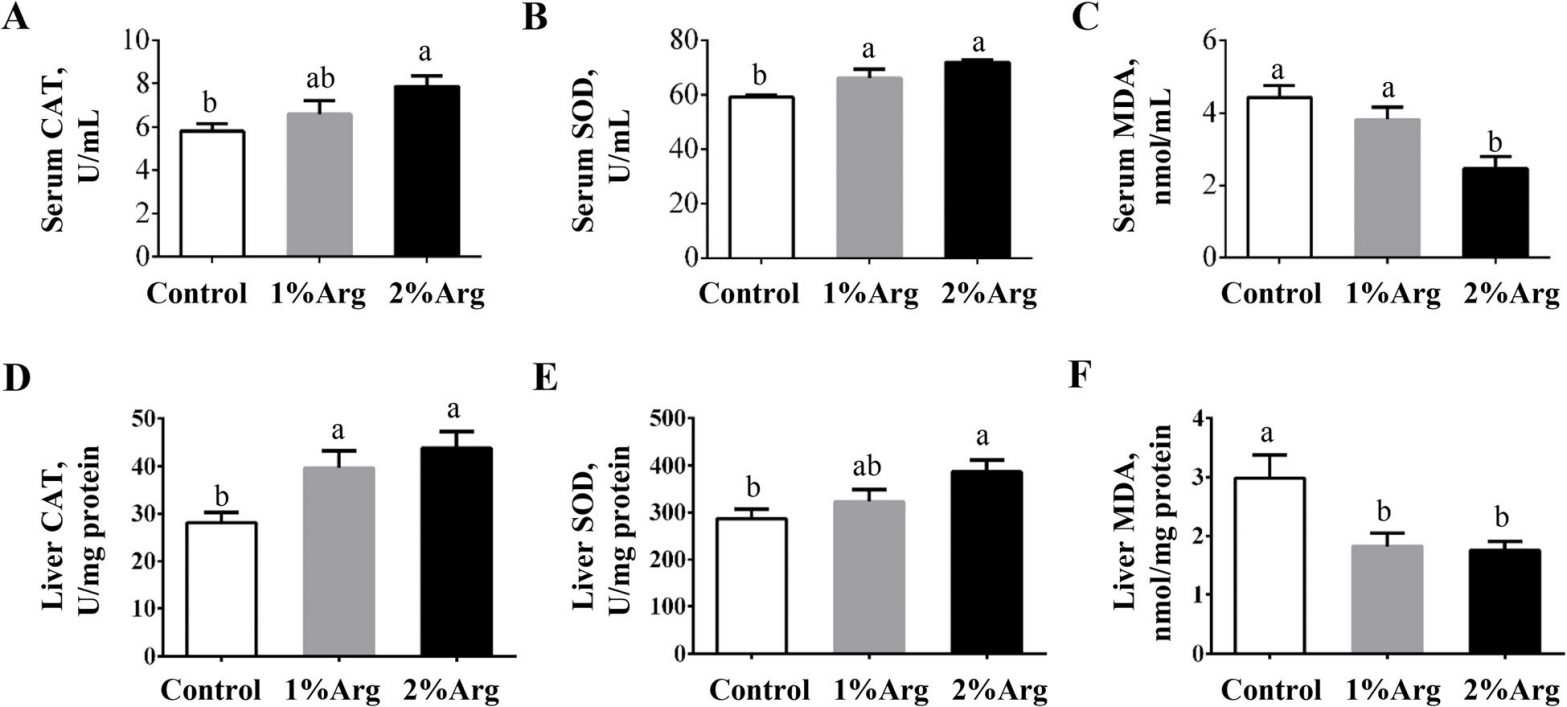
Activities of CAT (**a**) and SOD (**b**) and MDA content (**c**) in serum, activities of CAT (**d**) and SOD (**e**) and MDA content (**f**) in the liver of tilapia fed a commercial diet un-supplemented (control) or supplemented with 1% or 2% Arg. Values are means ± SEM, *n* = 3 tanks per group. Means without a common letter differ, *P* < 0.05. CAT, catalase; MDA, malondialdehyde; SOD, superoxide dismutase

Compared with the control group, the activity of serum CAT was significantly enhanced (*P* < 0.05) by dietary supplementation with 2% Arg, but not 1% Arg (Fig. 6a). Supplementation with 1% or 2% Arg significantly increased (*P* < 0.05) the activity of serum SOD (Fig. 6b). The concentration of MDA in serum was significantly decreased (*P* < 0.05) by supplementation with 2% Arg, but not 1% Arg (Fig. 6c). Additionally, supplementation with 1% or 2% Arg significantly augmented (*P* < 0.05) the hepatic activity of CAT (Fig. 6d), while significantly decreased (*P* < 0.05) the hepatic concentration of MDA (Fig. 6f). The hepatic activity of SOD was significantly enhanced (*P* < 0.05) by supplementation with 2% Arg, but not 1% Arg (Fig. 6e).

## Discussion

The liver is a major organ for lipid metabolism, and hepatic dysfunction is a major metabolic problem in fish that causes growth restriction and high costs of the aquaculture enterprise [8]. Based on the previous studies with mammals [14, 15], we investigated the role of dietary supplementation with Arg on intrahepatic fat accumulation and serum metabolic profiles in tilapia. We found that dietary Arg supplementation decreased lipid accretion and oxidative damage in the liver, as well as the diameter of adipocytes in intraperitoneal adipose tissue. In addition, Arg supplementation reduced the proportion of total MUFAs, while increasing the proportion of total PUFAs (both n-3 and n-6 PUFAs) in the liver and intraperitoneal adipose tissue. This beneficial effect of Arg was associated with decreases in mRNA levels for *Srebp-1*, *Accα*, *Scd*, and *Fas*, as well as increases in hepatic mRNA levels for *Cpt1α, Pparα*, *Δ4 Fads2,* and *Elovl5*. These results revealed a beneficial effect of dietary Arg supplementation on lipid metabolism in tilapia.

As in mammals [7], a high-fat diet was fed to tilapia to induce hepatic abnormalities. Specifically, the fish were fed a commercial diet containing 13% fat, which is higher than the crude fat of 5–7% in regular diets [13]. We found that Arg supplementation had no effect on feed intake, feed conversion ratio, or growth performance, but reduced intraperitoneal fat mass in tilapia and the size of adipocyte, as compared with the control group. It has been reported that a decrease in plasma lipid levels is associated with reduced risk of metabolic diseases [19]. Therefore, we determined serum lipid levels, including TG, TC, LDL, and HDL. Our results indicated reductions in serum concentrations of TG, TC, LDL, and HDL in Arg-supplemented fish, indicating a beneficial effect of Arg on improving their metabolic profiles. This result was consistent with previous studies in pigs [20], rats [14], and humans [15].

MUFA is less susceptible to be oxidized than PUFA, and are major fatty acids in the liver of freshwater fish [21, 22]. Consistently, lipid accumulation has been reported to be associated with decreased PUFA and increased MUFA in the livers of various animals [23, 24]. In our study, Arg supplementation reduced total MUFAs, and elevated total PUFAs, without affecting total SFAs in the liver and intraperitoneal adipose tissue. Considering that there was no difference in fatty acid composition among the three groups of diets, our results indicated a modulatory effect of Arg on fatty acid metabolism and profiles in the tissues of tilapia. PUFA biosynthesis involves multiple steps, including fatty acid desaturation and carbon chain extension, which were catalyzed by enzymes of *Fads* and *Elovl* family [25]. Here, we found that Arg supplementation increased mRNA levels for *Δ4 Fads2*, and *Elovl5*, without affecting those for *Δ5/Δ6 Fads*. The expression of *Fads* and *Elovl* is regulated by transcriptional factors such as *Pparα*, *Srebp-1*, *Lxr*, and *Hnf4α* in vertebrates [26, 27]. Consistent with this view, we found that Arg supplementation upregulated the mRNA level of *Pparα,* which binds to the promoter regions of *Δ4 Fads2* and *Elovl5,* therefore enhancing the mRNA levels of these two genes and contributing to PUFA syntheses in the liver of tilapia.

A salient observation from the present study is Arg supplementation reduced lipid concentrations and oxidative damage in the liver, as shown by (1) reductions in serum TG and TC, as well as oxidative damage and health markers (including serum ALT, AST, MDA, and LDH); and (2) increases in hepatic activities of SOD and CAT, two critical anti-oxidative enzymes implicated in the scavenging of reactive oxygen species and in hepatic homeostasis [28]. This result was consistent with previous studies in rats and other animals [29, 30].

Fat accretion in the liver and other tissues is dependent on a balance between fatty acid synthesis and oxidation. *Srebp-1* is a critical transcriptional factor related to fatty acid synthesis by activating downstream targets, including *Accα*, *Fas*, and *Scd* [31]. In contrast, *Cpt1α* and *Pparα* are key regulators of fatty acid oxidation [13]. Activation of *Srebp-1* or inhibition of *Cpt1α* and *Pparα* is associated with lipid accumulation in multiple tissues. Arg supplementation led to the downregulation of *Srebp-1*, *Accα*, *Fas*, and *Scd*, as well as upregulation of *Cpt1α* and *Pparα* in the liver, therefore contributing to reduced lipid accretion in the liver and intraperitoneal adipose tissue. Considering that Arg supplementation did not affect mRNA levels for *Cd36* and *Fatp5*, two transport proteins of long-chain fatty acids [13], our findings indicated that reduced lipid accumulation in the liver of tilapia was not due to a decrease in the hepatocytic uptake of fatty acids. In contrast, this modulatory effect of Arg was likely mediated by reduced lipogenesis and enhanced fatty acid oxidation, as evidenced by altered mRNA levels for key genes involved in these two biochemical processes. Further studies are required to uncover the underlying mechanisms responsible for the regulatory effect on tilapia.

## Conclusions

In conclusion, dietary supplementation with 1% or 2% Arg to tilapia reduced lipid accretion and oxidative damage in the liver, while increasing the proportion of total PUFAs, n-3 PUFAs, and n-6 PUFAs in the liver and intraperitoneal adipose tissue. These beneficial effects of Arg were associated with altered expression of key genes for lipid metabolism (including PUFA synthesis). Dietary Arg supplementation may provide an effective nutritional strategy to reduce excessive lipid accretion in fish as in mammals.

## Supplementary information

**Additional file 1 Supplemental Table 1** Ingredient composition and chemical analysis of the basal diet for tilapia **Supplemental Table 2** Amino acid composition of the basal diet un-supplemented (control), or supplemented with 1% Arg, or 2% Arg (g/kg diets) **Supplemental Table 3** Fatty acid composition (% total fatty acids) of the basal diet un-supplemented (control), or supplemented with 1% Arg, or 2% Arg for 56 days^1^**Supplemental Table 4** Quantitative PCR primers used in tilapia^1^**Supplemental Figure 1** Gross alteration of the liver and intraperitoneal fat from tilapia fed a basal diet un-supplemented (control), or supplemented with 1% Arg, or 2% Arg for 56 days.

## Data Availability

The datasets produced and/or analyzed during the current study are available from the corresponding author on reasonable request.

## Abbreviations

*Accα*: Acetyl-CoA carboxylase *α*;
Ala: *L*-Alanine
ALT: Alanine aminotransferase
Arg: *L*-Arginine
AST: Aspartate aminotransferase
CAT: Catalase
*Cd36*: Cluster determinant 36
*Cpt1α*: Carnitine palmitoyltransferase 1*α*
*Elovl5*: Elongase 5 of very long-chain fatty acids
*Fads2*: Fatty acyl desaturase 2
*Fas*: Fatty acid synthase
*Fatp5*: Fatty acid transport protein 5
HDL: High-density lipoprotein cholesterol
*Hnf4α*: Hepatocyte nuclear factor 4α
LDH: Lactate dehydrogenase
LDL: Low-density lipoprotein cholesterol
Lxr: Liver X receptor
MDA: Malondialdehyde
MUFA: Monounsaturated fatty acid
NEFA: Non-esterified free fatty acids
Pparα: Peroxisome proliferator-activated receptor *α*
PUFA: Polyunsaturated fatty acid
Scd: Stearoyl-CoA desaturase
SOD: Superoxide dismutase
Srebp-1: Sterol regulatory element-binding protein-1
TC: Total cholesterol
TG: Triglycerides
