# Supplementary material for: Dietary L-arginine supplementation reduces lipid accretion by regulating fatty acid metabolism in Nile tilapia (Oreochromis niloticus)
Source: J Anim Sci Biotechnol. 2020 Aug 14;11:82. doi: 10.1186/s40104-020-00486-7 (PMC7427058; doi:10.1186/s40104-020-00486-7)
Supplement: Supplementary file 1 — Additional file 1 Supplemental Table 1 Ingredient composition and chemical analysis of the basal diet for tilapia Supplemental Table 2 Amino acid composition of the basal diet un-supplemented (control), or supplemented with 1% Arg, or 2% Arg (g/kg diets) Supplemental Table 3 Fatty acid composition (% total fatty acids) of the basal diet un-supplemented (control), or supplemented with 1% Arg, or 2% Arg for 56 days1Supplemental Table 4 Quantitative PCR primers used in tilapia1Supplemental Figure 1 Gross alteration of the liver and intraperitoneal fat from tilapia fed a basal diet un-supplemented (control), or supplemented with 1% Arg, or 2% Arg for 56 days. [file 40104_2020_486_MOESM1_ESM.docx]

**Supplemental Table 1** Ingredients composition and chemical analysis of the basal diet for tilapia

| Ingredients | Percentage (%; as-fed basis) |
| --- | --- |
| Cottonseed meal | 10 |
| Fish meal | 10 |
| Rapeseed meal | 14 |
| Rice bran | 14 |
| Soybean meal | 22 |
| Soybean oil | 9 |
| Wheat bran | 10 |
| Wheat flour | 10 |
| Vitamin and mineral premix^1^ | 1 |
| Proximate composition, g/kg diet |  |
| Dry matter | 900 |
| Crude protein | 313 |
| Crude lipid | 130 |
| Ash | 80 |
| Gross energy, kcal/g diet | 10 |

^1^Providing the following per kilogram of diet: 18 mg of calcium pantothenate; 150 mg of choline chloride; 0.02 mg of Co; 3 mg of Cu; 80 mg of Fe; 1 mg of folic acid; 0.04 mg of I; 80 mg of inositol; 330 mg of Mg; 10 mg of Mn; 18 mg of nicotinic acid; 0.03 mg Se; 7000 IU of vitamin A; 11 mg of vitamin B_1_; 10 mg of vitamin B_2_; 6 mg of vitamin B_6_; 0.025 mg of vitamin B_12_; 80 mg of vitamin C; 500 IU of vitamin D_3_; 40 mg of vitamin E; 4 mg of vitamin K_3_; 30 mg of Zn.

**Supplemental Table 2** Amino acid composition of the basal diet un-supplemented (control), or supplemented with 1% Arg, or 2% Arg (g/kg diets)

| Amino acid | Control | 1%Arg | 2%Arg |
| --- | --- | --- | --- |
| *L*-Aspartate + *L*-Asparagine | 29.34 | 29.06 | 28.55 |
| *L*-Alanine | 60.04 | 41.79 | 19.37 |
| *L*-Arginine | 16.90 | 31.03 | 45.08 |
| *L*-Glutamate + *L*-Glutamine | 57.00 | 59.42 | 61.50 |
| Glycine | 11.63 | 11.88 | 12.16 |
| *L*-Histidine | 7.29 | 7.82 | 8.53 |
| *L*-Isoleucine | 12.99 | 13.04 | 13.05 |
| *L*-Leucine | 21.94 | 22.01 | 22.13 |
| *L*-Lysine | 11.88 | 11.87 | 11.83 |
| *L*-Methionine | 4.31 | 4.24 | 4.21 |
| *L*-Phenylalanine | 14.89 | 14.97 | 15.00 |
| *L*-Serine | 14.52 | 15.68 | 16.42 |
| *L*-Threonine | 10.17 | 10.75 | 11.28 |
| *L*-Tyrosine | 9.08 | 9.12 | 9.22 |
| *L*-Tryptophan | 1.99 | 2.00 | 2.05 |
| *L*-Valine | 16.41 | 16.42 | 16.64 |

**Supplemental Table 3** Fatty acid composition (% total fatty acids) of the basal diet un-supplemented (control), or supplemented with 1% Arg, or 2% Arg for 56 days^1^

| Fatty acid | Control | 1%Arg | 2%Arg |
| --- | --- | --- | --- |
| C12:0 | 0.23 | 0.16 | 0.17 |
| C14:0 | 0.59 | 0.57 | 0.58 |
| C15:0 | 0.05 | 0.05 | 0.06 |
| C16:0 | 13.94 | 13.75 | 13.85 |
| C17:0 | 0.13 | 0.13 | 0.13 |
| C18:0 | 3.82 | 3.88 | 3.87 |
| C20:0 | 0.40 | 0.42 | 0.41 |
| C21:0 | 0.06 | 0.07 | 0.06 |
| C22:0 | 0.38 | 0.40 | 0.39 |
| C23:0 | 0.09 | 0.09 | 0.09 |
| C24:0 | 0.36 | 0.37 | 0.36 |
| ∑ SFAs | 20.06 | 19.90 | 19.96 |
| C16:1n-7 | 0.73 | 0.71 | 0.72 |
| C18:1n-9 | 24.71 | 24.86 | 24.82 |
| C20:1n-9 | 0.33 | 0.34 | 0.34 |
| C22:1n-9 | 0.07 | 0.07 | 0.07 |
| C24:1n-9 | 0.06 | 0.07 | 0.07 |
| ∑ MUFAs | 25.89 | 26.05 | 26.01 |
| C18:3n-3 | 5.23 | 5.22 | 5.21 |
| C20:5n-3 | 0.80 | 0.78 | 0.78 |
| C22:6n-3 | 0.66 | 0.63 | 0.64 |
| ∑ n-3 PUFAs | 6.69 | 6.63 | 6.63 |
| C18:2n-6 | 47.26 | 47.31 | 47.28 |
| C20:2n-6 | 0.01 | 0.02 | 0.02 |
| C20:4n-6 | 0.09 | 0.09 | 0.09 |
| ∑ n-6 PUFAs | 47.36 | 47.43 | 47.39 |
| ∑ PUFAs | 54.05 | 54.06 | 54.02 |
| n-6:n-3 | 7.08 | 7.15 | 7.15 |

^1^MUFAs, monounsaturated fatty acids; n-6:n-3, n-6 PUFAs:n-3 PUFAs; PUFAs, polyunsaturated fatty acids; SFAs, saturated fatty acids.

**Supplemental Table 4** Quantitative PCR primers used in tilapia^1^

| Genes | Sequences (5’ to 3’) | Product size, bp | GenBank No. |
| --- | --- | --- | --- |
| *Δ6/Δ5 Fads2* | F: GTGGATCTGGCTTGGTTCAT | 186 | XM_031747436.1 |
|  | R: CCAGTCCCTGTGCTTTTCAT |  |  |
| *Δ4 Fads2* | F: CTTACTGTGCTCGGTGATT | 209 | XM_003440472 |
|  | R: GGTCCTTGCTGAAGATGTT |  |  |
| *Accα* | F: TAGCTGAAGAGGAGGGTGCAAGA | 110 | XM_025910659.1 |
|  | R: AACCTCTGGATTGGCTTGAACA |  |  |
| *Cd36* | F: GGGCATCTTCAGAGATCGCA | 160 | XM_003452029.5 |
|  | R: GGGTTGAATGTGACGTTCGC |  |  |
| *Cpt1α* | F: TTTCCAGGCCTCCTTACCCA | 102 | XM_031737259.1 |
|  | R: TTGTACTGCTCATTGTCCAGCAGA |  |  |
| *Elovl5* | F: GCCATACCTTTGGTGGAAGA | 228 | XM_031742894.1 |
|  | R: AGGGAGCTGTTCTGTGGATG |  |  |
| *Ef1α* | F: GCACGCTCTGCTGGCCTTT | 250 | XM_019350343.1 |
|  | R: GCGCTCAATCTTCCATCCC |  |  |
| *Fas* | F: TGAAACTGAAGCCTTGTGTGCC | 141 | XM_003454056.5 |
|  | R: TCCCTGTGAGCGGAGGTGATTA |  |  |
| *Fatp5* | F: TACACATCTGGGACCACAGGTTTG | 110 | XM_003443859.5 |
|  | R: AAGATGTCCTCTGCTGTGACTCCA |  |  |
| *Hnf4α* | F: AACGAGACAGAATCAGCACC | 206 | XM_003457051.5 |
|  | R: CCACTCCACTAAGACCAACAG |  |  |
| *Lxr* | F: GTAAGGTGTTTGATGGGGC | 143 | XM_005455714.4 |
|  | R: ATTATGAGGGGGGACGG |  |  |
| *Pparα* | F: TGGTTCGGGGTCCAATAG | 247 | XM_031750632.1 |
|  | R: GCAGTTCCGCTCACACTTAT |  |  |
| *Scd* | F: ACAAGCTCTCCGTGCTGGTCAT | 102 | XM_005471382.2 |
|  | R: GCAGAGTTGGGACGAAGTAGGC |  |  |
| *Srebp-1* | F: TGCAGCAGAGAGACTGTATCCGA | 102 | XM_005457771.4 |
|  | R: ACTGCCCTGAATGTGTTCAGACA |  |  |

^1^*Accα*, acetyl-CoA carboxylase α; *Cd36*, cluster determinant 36; *Cpt1α*, carnitine palmitoyltransferase 1α; *Elovl5*, elongase 5 of very long-chain fatty acids; *Fads2*, fatty acyl desaturase 2; *Fas*, fatty acid synthase; *Fatp5*, very long-chain acyl-CoA synthetase 5; *Hnf4α*, hepatocyte nuclear factor 4α; *Lxr*, liver x receptor; *Pparα*, peroxisome proliferator activated receptor α; *Scd*, acyl-CoA desaturase; *Srebp-1*, sterol regulatory element-binding protein 1.

**Supplemental Figure 1** Gross inspection alteration of the liver and intraperitoneal fat from tilapia fed a basal diet un-supplemented (control), or supplemented with 1% Arg, or 2% Arg for 56 days.

**
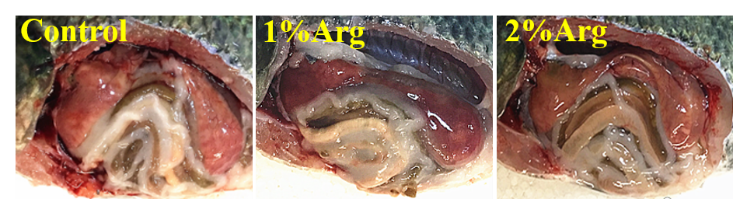
**
